# Deciphering shared receptor usage in genomically unrelated bacteriophages infecting hypervirulent *Klebsiella pneumoniae* K1 ST23

**DOI:** 10.1093/femsmc/xtaf014

**Published:** 2025-10-20

**Authors:** Zhanybek Selpiev, Sebastian Leptihn, Mathias Müsken, Belinda Loh

**Affiliations:** Fraunhofer Institute for Cell Therapy & Immunology (IZI), Department of Infection Research & Diagnostics, Perlickstr. 1, 04103 Leipzig, Germany; Institute of Biology, Leipzig University, Talstr. 33, 04103 Leipzig, Germany; Department of Biochemistry, Health and Medical University Erfurt, Anger 66/73, 99084 Erfurt, Germany; Department of Biochemistry and Molecular Biology, University of Southern Denmark, Campusvej 55, 5230 Odense, Denmark; Central Facility for Microscopy, Helmholtz Centre for Infection Research, Inhoffenstr. 7, 38124 Braunschweig, Germany; Fraunhofer Institute for Cell Therapy & Immunology (IZI), Department of Infection Research & Diagnostics, Perlickstr. 1, 04103 Leipzig, Germany

**Keywords:** *Klebsiella pneumoniae* ST23, bacteriophage, capsular polysaccharide (CPS), host–phage interaction, tail fiber structural homology, genetic mosaicism

## Abstract

*Klebsiella pneumoniae* is a critical pathogen often associated with multidrug resistance and hypervirulence. We report the isolation and characterization of three distinct lytic bacteriophages—Spear, Loop, and Shorty—from sewage, using a hypervirulent, hypermucoid *K. pneumoniae* K1 ST23 strain as the host. Despite genomic and structural differences, all three phages exhibited a narrow host range, infecting only the K1 serotype. Transmission electron microscopy and genomic analyses confirmed their lytic lifestyle and classifications: Spear and Loop are siphovirus-like, while Shorty is podovirus-like. A key focus was phage–host interaction and receptor usage. DNA sequence analysis showed no homology between the receptor-binding proteins, yet structural modelling revealed high similarity between Loop and Shorty tail fibers, aligning within a K1-specific lyase domain, suggesting phage genetic mosaicism. All three phages rely on capsular polysaccharide (CPS) for infection. Resistance selection under phage pressure yielded non-mucoid mutants, characteristic of CPS loss. Cross-resistance and adsorption assays confirmed CPS-dependence. Loop and Shorty showed near-complete loss of binding; Spear retained partial binding, suggesting additional receptors. These results highlight that unrelated phages can target the same bacterial structure, CPS. This has important implications for rational phage cocktail design, as CPS mutations may undermine seemingly diverse phage combinations.

## Teaser

Capsular polysaccharide is identified as a shared receptor among diverse bacteriophages infecting *Klebsiella pneumoniae* K1 ST23, offering new insights for rational phage therapy design.

## Introduction

*Klebsiella pneumoniae* is a Gram-negative bacterium causing a spectrum of human infections, including pneumonia, bloodstream, urinary tract, and wound infections, and is a leading cause of healthcare-associated infections worldwide (Podschun and Ullmann 1998, Martin and Bachman 2018, Ding et al. 2023). The epidemiology of *Klebsiella* is characterized by its remarkable adaptability and widespread distribution (Wyres et al. 2020). Beyond its role as a human pathogen, *Klebsiella* also colonizes a variety of animals, including livestock and companion animals, and exhibits a notable ability to persist in diverse environmental niches such as soil and water, independent of an animal or human host (Podschun and Ullmann 1998, Davis and Price 2016). This environmental resilience contributes to its dissemination and is a prime example for the interconnectedness of human, animal, and environmental health, underscoring the importance of ‘One Health’ approach with regards to this pathogen (McEwen and Collignon 2018, Wyres et al. 2020).

Contributors to the pathogenicity of *K. pneumoniae* are the high degree of capsule diversity, with numerous capsular serotypes contributing to variations in virulence and immune evasion (Lee et al. 2021, Huang et al. 2022, Zierke et al. 2025, Liu et al. 2025). In addition to the observed increasing rates of antibiotic resistance, hypermucoviscosity adds another layer of complexity. Hypermucoviscosity is characterized by an overexpression of capsular polysaccharide (CPS) on the surface of bacteria, which protects bacterial communities from both antibiotic efficacy and host immune responses (Chen et al. 2024, Zierke et al. 2025). Consequently, treatment of *Klebsiella* infections is becoming increasingly challenging, driving the exploration of alternative therapeutic strategies.

One such promising approach is phage therapy, which utilizes viruses that specifically infect and kill bacteria, to target and eliminate pathogens (Strathdee et al. 2023). Despite its potential for precise bacterial targeting, phage therapy faces substantial challenges, particularly in eliminating *Klebsiella* strains due to their diverse capsule composition (Haudiquet et al. 2024). These difficulties stem from the inherent high specificity of phages and the rapid development of phage resistance (Nang et al. 2024). To effectively target *Klebsiella*, large and diverse phage collections are required, as individual phages generally display narrow host ranges (Strathdee et al. 2023).

Despite the diversity of *Klebsiella* phages, there is insufficient data about the receptor usage of phages infecting hypervirulent K1 strains, and how this may impact the evolution of resistance and phage therapy. In this study, we present three genomically unrelated phages—Shorty, Spear, and Loop—that infect the same hypervirulent and hypermucoid *K. pneumoniae* K1 ST23 strain. We investigate their biological characteristics, such as morphology and genomic features, along with in-depth analysis into their receptor usage and receptor-binding domains. A critical aspect of our work is the examination of the shared receptor usage among these phages, specifically of the CPS. This shared usage, despite distinct phage classifications (siphovirus-like and podovirus-like) and genomic sequences, suggests a common evolutionary pressure on bacterial targets, which in turn reveals that relying solely on phage family diversity or sequence differences does not guarantee broad efficacy in phage cocktail design. Furthermore, the domains containing the receptor-binding entities do not only show sequence homology but are also structurally almost identical. This observed structural homology, particularly given the otherwise distinct genomic backgrounds, indicates genetic mosaicism as a mechanism for the acquisition of K1-specific receptor recognition elements. Our findings emphasize the importance of understanding phage–host interactions and their implications for bacterial virulence attenuation upon resistance acquisition. It also advocates for a move beyond traditional empirical phage selection, which often relied on morphological assessment, towards a more rational and receptor-guided approach to developing robust phage therapeutics against prevalent pathogens like hypervirulent *K. pneumoniae*.

## Materials & methods

### Strains and environmental samples

All *K. pneumoniae* strains were obtained from the University Hospital in Leipzig, Germany. The isolation host (Kp290-019) is a hypermucoid and hypervirulent strain of ST23 K1 lineage. Sewage water was collected from three different sewage treatment plants within the state of Saxony, Germany. Three separate sample pools were created. Each sample pool consisted of 10 different samples collected at the same sewage treatment plant. The sample pools were centrifuged at 3700 × *g* for 15 min. After centrifugation, samples were filtered through a 0.45 µm PES filter and stored at 4°C until further use.

### Phage isolation and purification

The *K. pneumoniae* culture was grown from a single colony in Lysogeny Broth (LB) at 37°C, 220 rpm overnight. The overnight culture was then mixed with an environmental sample in a 1:1 ratio. The resulting culture was incubated over a period of 16 h at 37°C, 220 rpm and was then centrifuged at 16 000 × *g* for 10 min. Chloroform was added to the supernatant at 0.05% (v/v) and centrifuged at 16 000 × *g* for 15 min. The top liquid phase was used for phage purification.

Phages were purified using the soft-agar overlay method at least three times. Briefly, a 10-fold serial dilution of the bacterial-environmental sample was made using LB medium. Top agar (0.7%) was mixed with a bacterial culture at OD_600_ = 0.6 at 10% (v/v) and a phage sample at 1% (v/v). The mixture was then poured onto an LB agar plate, and allowed to solidify. The plates were incubated at 37°C over a period of 16 h. The resulting single plaques on the plate were then isolated with a Pasteur pipette and deposited into LB medium and left at 4°C (cold room) for 16 h. This procedure was repeated until uniform plaques were obtained.

### Phage screening

Different *K. pneumoniae* strains were grown as liquid cultures until OD_600_ = 0.6 was reached. The respective liquid cultures were mixed with 0.7% top agar at 10% (v/v) and overlayed on pre-made LB plates. Phage samples were spotted on the agar surface after the top agar had solidified, and incubated at 37°C for 12 h. Post incubation, the plates were observed for the presence of plaques.

### Phage titering

A 10-fold serial dilution of the purified phage sample was made up to a 10^9^ dilution. Phages were plated as described above. Only plates displaying well-isolated single plaques were used for plaque counting. Phage titer (PFU/ml) was derived based on the number of plaques, sample dilution and sample volume. Efficiency of plating was determined following the same procedure, but using different *K. pneumoniae* strains instead (other than the original host).

### Host range determination

The host range was assessed using 48 clinical *K. pneumoniae* strains. While more than 130 capsule (K) types exist, our strain collection comprises only three K1 isolates (only 2 of which are K1 ST23), alongside others including K2, K3, K14/K64, K18, K19, K20, K22/37, K23/K52, K24, K25, K30, K41, K55, K60, K62, as well as several strains with unknown K-types. Each purified phage sample was spotted onto the plates with different strains. Any phage-strain combination that resulted in clearance of bacterial lawn was recorded. The experiment was repeated twice for only the positive phage-strain combinations using top agar overlay and phage streaking techniques.

### Genome isolation

The purified phage sample was incubated with 10% (v/v) 10x DNase I buffer, 0.2% v/v DNase I (1 U/µl) and 0.2% v/v RNase A (10 mg/ml) at 37°C for 90 min. EDTA was added to reach a final concentration of 20 mM and incubated at 75°C for 15 min. The proteinase K (20 mg/ml) was added at 0.4% alongside 10% SDS added at 9% (v/v). The solution was incubated without shaking at room temperature over a period of 12 h. An equal volume of ROTI^®^Phenol/chloroform/isoamyl alcohol was added and mixed gently by inverting. The mixture was centrifuged in the benchtop centrifuge at 16 000 × *g* for 10 min. Chloroform was added to the top aqueous phase in the 1:1 volume ratio, and gently mixed. The mixture was centrifuged again at 16 000 × *g* for 10 min. A 3 M of sodium acetate was added to the top aqueous phase at 10% (v/v) together with 100% ethanol added at a volume ratio of 2.5:1. The solution was incubated at −20°C for 12 h and then centrifuged at 16 000 × *g* for 10 min in the benchtop centrifuge. Finally, the pellet was rinsed with 70% ethanol, dried, dissolved in Milli-Q^®^ water, and stored at −20°C. Whole genome sequencing was done via Illumina sequencing.

### *In silico* phage sequence analyses

Sequencing files were adapter-trimmed using Trim Galore v0.6.10 program. The resulting files were normalized and error-corrected using BBnorm function of BBMap v38.98 (sourceforge.net/projects/bbmap/). The genomes were assembled using SPAdes v3.15.3 and visualized in Bandage v0.9.0 (Wick et al. 2015, Prjibelski et al. 2020). Quality of the assembled genomes was controlled through Minimap2 v2.22 and SAMtools v1.14 package (Li 2018, Danecek et al. 2021). Genome annotation was conducted using pharokka v1.7.3 against Prokaryotic virus Remote Homologous Groups (PHROGs), the Comprehensive Antibiotic Resistance Database (CARD), and Virulence Factor Database (VFDB) databases (Bouras et al. 2023). Genome annotations were further improved with phold v0.1.4 and phynteny v0.1.13 rapid annotation tools to assign gene functions based on structure homology and synteny (Grigson, 2023). Phage life cycles were predicted via BACPHLIP v0.9.3-alpha tool using assembled genomes as an input (Hockenberry and Wilke 2021).

Genome maps were created using plot function of phold v0.1.4 tool. Phage genomes were aligned in NCBI nucleotide BLAST (blast.ncbi.nlm.nih.gov) against non-redundant database filtered to Caudovirales (taxid:2 731 619). BLAST results were used to construct phylogenetic tree using VICTOR online tool (Meier-Kolthoff and Göker 2017). Phylogenetic trees were customized in iTOL v6 online tool (Letunic and Bork 2024).

Putative tail fiber sequences were aligned to each other in NCBI nucleotide and protein BLAST. The sequences were additionally searched for remote homology using HHPred web server (Zimmermann et al. 2018) against the following three databases: PDB_mmCIF70_30_Mar, UniProt-SwissProt-viral70_3_Nov_2021, and NCBI_Conserved_Domains(CD)_v3.19. Tail fiber structure predictions were generated as trimers on AlphaFold3 server (Abramson et al. 2024). The trimeric structures were tested for structure-homology against each other using TM-align server (Zhang and Skolnick 2005). Predicted homologous structures were superimposed onto each other in Chimerax v1.9 using Matchmaker function (Meng et al. 2023).

### Transmission electron microscopy

Phages were amplified using the soft-agar overlay assay at MOI of 1, incubated at 37°C for 6 h. To each bacterial plate, 2 ml of phage buffer (10 mM Tris–HCl, pH 7.5, 10 mM MgSO_4_, 1 mM CaCl_2_, 68 mM NaCl) was added and incubated at room temperature, shaking at 30 rpm for 5 h. Phage containing buffer was collected and passed through a 0.22 μm filter. Filtrate was then centrifuged at 20 000 × *g* for 1 h at 4°C. Supernatant was discarded leaving the last 20 μl to 50 μl. Phage pellet was then resuspended with 100 μl water and stored at 4°C. The phage samples were adsorbed onto thin carbon support films, followed by negative staining with a 2% (w/v) aqueous uranyl acetate solution (pH 5.0). Examination of the samples were conducted with either a Zeiss EM 910 or Zeiss Libra120 Plus transmission electron microscope (Carl Zeiss, Oberkochen, Germany) at an acceleration voltage of 80 kV/120 kV and at calibrated magnifications. Size determination of heads and tails was performed using ITEM Software (Olympus Soft Imaging Solutions, Münster).

### Thermal stability

Phage samples at a titer of 10^9^ PFU/ml were exposed to 8 temperatures from 20°C to 80°C. Each sample was incubated at the corresponding temperature for 1 h prior to making a 10-fold dilution series. Phage titer of each sample was then determined via soft agar overlay.

### Resistant variant generation

Bacterial culture at OD_600_ = 0.6 was infected with phages at a final MOI of 10 and overlayed in LB soft-agar onto an LB agar plate. The plate was incubated at 37°C over a period of 16 h. Twenty-five distinct colonies were picked and streaked on a set of LB agar plates and incubated at 37°C for 16 h. Resulting colonies were propagated onto a new set of plates to produce uniform bacterial populations.

### Imaging colony morphologies

Mucoid and non-mucoid colonies were visualised using ZEISS SteREO Discovery.V8 microscope with ZEISS CL 6000 LED as cold light source. Images were taken with a rear camera of an Apple iPad 9th generation (MK2L3FD/A). Final image were generated by overlaying two microscopy images captured from axial and lateral views of the same specimen.

### Adsorption assay

Bacterial cultures were inoculated from single colonies and grown at 37°C, 220 rpm until an OD_600_ = 1.0 was reached. Phage samples at a titer of 10^9^ PFU/ml were added to the bacterial cultures at 10% (v/v). Bacteria-phage samples were incubated on ice for 20 min. The tubes were centrifuged at 16 000 × *g*, 4°C for 5 min. The supernatant was transferred, and a 10-fold dilution series was made. The titer of the supernatant was determined via soft-agar overlay technique.

## Results

### Three distinct phages infect *Klebsiella pneumoniae*

**Figure 1.**
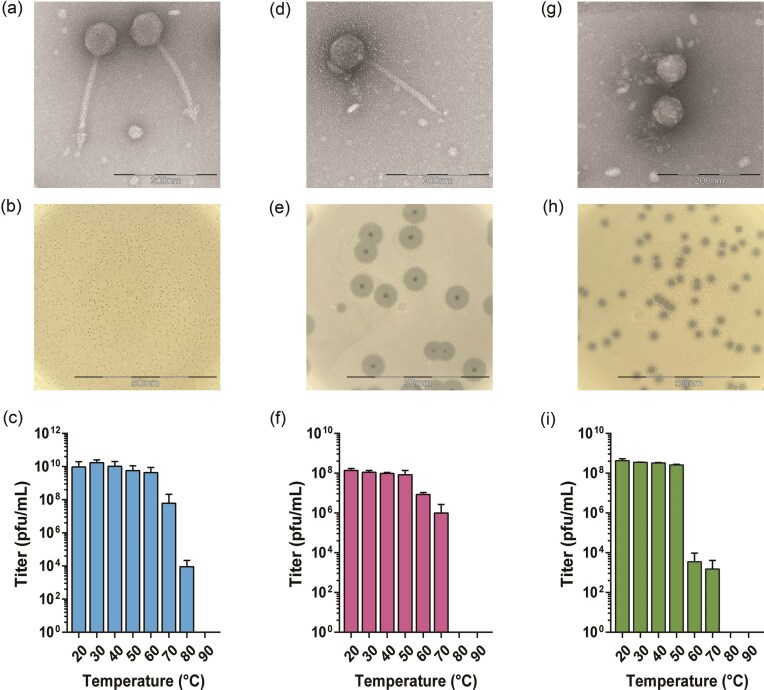
Three distinct bacteriophages infect ST23 *K. pneumoniae* strain. (a, d, g) TEM images of phages. (b, e, h) Plaque morphologies of each phage on LB plates. Phage Spear has a siphovirus-like structure (a) which generates small pin-prick plaques (b). Phage Loop has a siphovirus-like structure (d) and it generates small plaques with broad ‘halos’ (e). Phage Shorty has a podovirus-like structure (g) and it generates big plaques with narrow ‘halos’ (h). (c, f, i) Thermostability of Spear, Loop, and Shorty.

Three bacteriophages, Shorty, Spear, and Loop, were isolated from sewage water samples, making use of *K. pneumoniae* as the host bacterial strain. The plaques obtained were purified by the double agar overlay method until each isolate was uniform on agar plates. These 3 bacteriophages were regarded as separate isolates due to their distinct plaque morphologies (Fig. 1b, e, and h). Phage Spear forms small clear plaques while Loop and Shorty form a halo surrounding the plaque, suggesting the presence of a depolymerase. Transmission electron microscopy (TEM) images of the purified phages reveal that both Spear and Loop are siphovirus-like particles with non-contractile tails. Despite this similarity, subtle differences in their structural detail suggest that they are indeed two distinct phages (Fig. 1a and d). Spear has a capsid of ~65 nm in length and 71 nm wide, and a tail of ~171 nm in length. In contrast, Loop has a smaller capsid (62 nm long, 68 nm wide) and a shorter tail (166 nm). However, perhaps the most striking difference can be found in the tail fibers where Spear features a distinct arrow head-like structure while Loop has a loop-like structure (Fig. 1a vs. 1d). Meanwhile, TEM images show Shorty as a podovirus-like phage characterized by a short non-contractile tail (Fig. 1g). Its average capsid dimensions are 62 nm by 63 nm but it has a tail that is poorly visible by negative staining.

Another parameter we examined to further evaluate their therapeutic potential was their host range; out of 48 *K. pneumoniae* strains tested via duplicate top agar overlay assays, all three phages infected both *K. pneumoniae* K1 ST23, O1ab serotype strains in our collection, the original isolating host and one other hypervirulent strain, indicating an extremely narrow host range.

Thermostability tests, used as a proxy for long term stability (e.g. for storage until deployment or persistence in a wound or the infected tissue), indicate that Spear, Loop, and Shorty retained infectivity up to 50°C, with a marked decline at higher temperatures (Fig. 1c, f, and i). Spear lost activity completely at 90°C, while Loop and Shorty were completely inactive by 80°C. Their stability supports the suitability of the phages for therapeutic applications.

### Genomic analysis of the unrelated phages Spear, Loop, and Shorty

**Figure 2.**
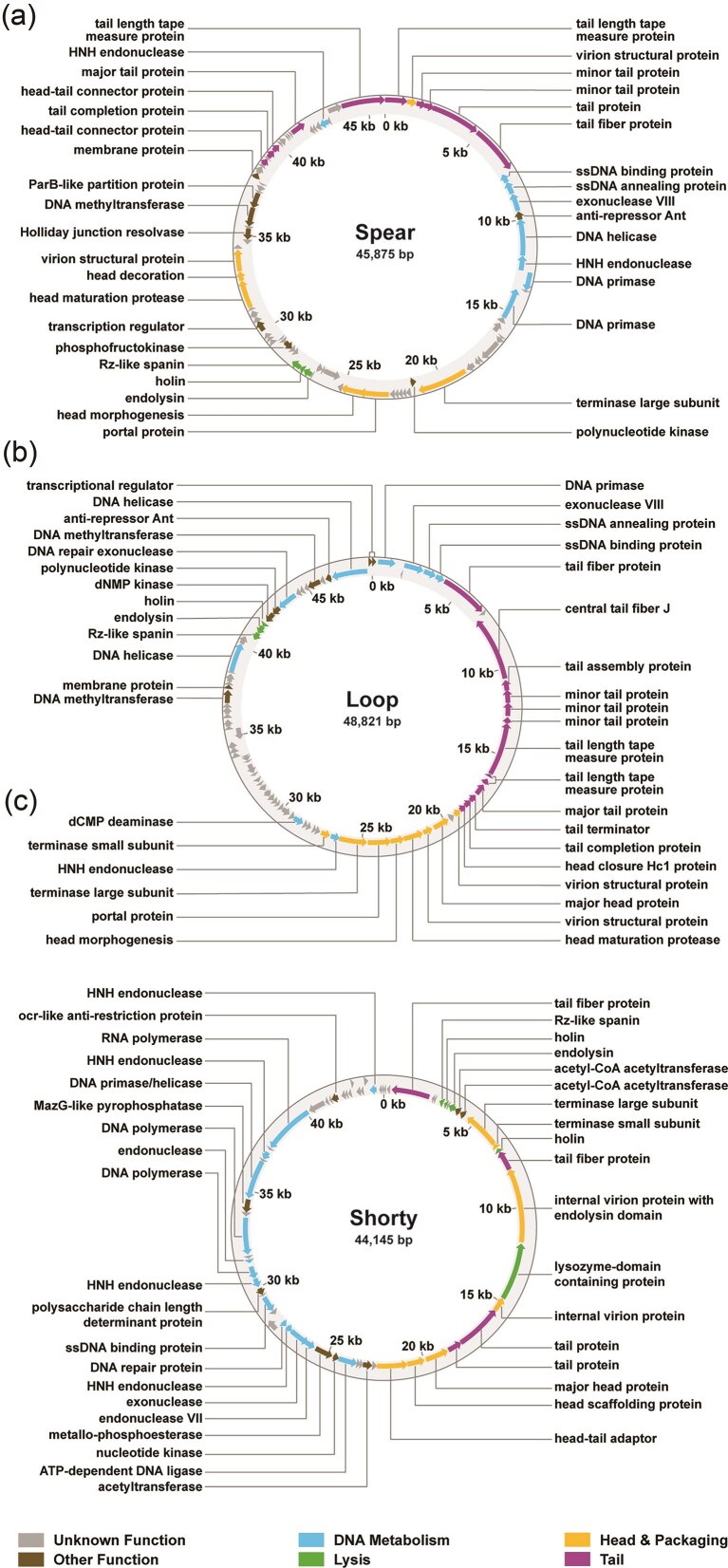
Annotated genomic map of phages Spear (a), Loop (b) and Shorty (c). Arrows dictate direction of transcription.

Whole genome sequencing was conducted to investigate phages Shorty, Spear and Loop bioinformatically. All three are double-stranded DNA (dsDNA) phages with genome sizes of 45 875 bp for Spear, 48 821 bp for Loop, and 44 145 bp for Shorty (Fig. 2). Annotation of the phage genomes identified 84 open-reading frames (ORFs) in Spear (43 [51%] of which are predicted hypothetical proteins) (Fig. 2a), 80 ORFs in Loop (with 38 [48%] genes of unknown function) (Fig. 2b), and 65 ORFs in Shorty (38 [58%] align to genes of known functions, while 27 [42%] remain functionally uncharacterized). Across the three phage genomes, the ORFs annotated with putative functions can be broadly classified into four main categories: (1) phage capsid and DNA packaging, (2) tail structure, (3) DNA metabolism, and (4) host cell lysis. Most functionally classified ORFs are predicted to mediate the assembly of infectious phage particles and support the efficient production of progeny viruses. Comparative analysis using nBLAST demonstrated that these genomes share no significant similarity among them. However, each phage exhibits homology to other phages within the NCBI BLAST database (Supp. Figs S1A, S2A, S3A).

Spear is most closely related to *Klebsiella* phage BUCT541 (NC_071154.1) (Pu et al. 2022), which also infects a *K. pneumoniae* K1 ST23 strain, sharing 77% query coverage and 96.89% nucleotide identity. Tail fiber proteins are 94.25% related to each other on the amino acid level. The genome size of phage BUCT541 (46 100 bp) is only 225 bp longer than that of Spear, prompting further examination of non-conserved genomic regions that underlie the remaining 23% non-aligned sequence. While the majority of genes are either identical or display only single nucleotide polymorphisms, including those involved in head morphogenesis and tail assembly, several open reading frames (ORFs) are unique to Spear. Notably, Spear encodes a predicted Rz-like spanin that is absent in BUCT541. BLASTp analysis revealed this spanin shares 99% identity with the corresponding protein from *Klebsiella* phage YX3973 (NC_054652.1) (Supp. Fig. S1B). Additionally, the endolysin from Spear is only 79.31% identical to its phage BUCT541 homologue, but shows greater similarity to the endolysin from *Klebsiella* phage RCIP0071 (OR532865.1). These differences within the lysis module suggests possible specialization or recent gene exchange events.

Loop was found to be highly similar to *Klebsiella* phage vB_KpnP_Henu1_3 (PQ133004.1), with 98% query coverage and 97.98% identity (Supp. Figs S1B, S2B), while Shorty displayed closest homology to *Klebsiella* phage Kpp-61 (PQ570679.1), with 94% query coverage and 96.94% identity (Supp. Fig. S3B). Further analysis of the tail fiber and tail spike proteins, crucial for host specificity, provided additional insights. Shorty’s tail fiber protein was 98.23% identical to that of Kpp-61, while its tail spike protein showed 96.14% identity to the Kpp-61 homologue, indicating that this phage is likely to also infect a K1 type *K. pneumoniae* strain. Indeed, source information detailed on the NCBI database indicate *K. pneumoniae* type K1 as the host of phage Kpp-61. For Loop, the tail fiber protein from vB_KpnP_Henu1_3 shares 99.84% identity, while the tail spike protein is 99.29% identical to its counterpart in vB_KpnP_Henu1_3, also indicating that phage vB_KpnP_Henu1_3 possibly targets *K. pneumoniae* K1 type strain. However, details disclosed on the host of phage vB_KpnP_Henu1_3 on NCBI indicate *K. pneumoniae* ATCC 700603, which is not a K1 strain but a K6 strain.

Interestingly, both siphovirus-like phages, Spear and Loop, appear to encode an anti-repressor protein (Ant), which could initially suggest a lysogenic life cycle (Lemire et al. 2011, Kim and Ryu 2013, Baaziz et al. 2024) (Fig. 2). However, none of the genomes contain integrase or repressor genes, which are hallmarks of a lysogenic life cycle. Furthermore, whole-genome sequencing of the *K. pneumoniae* host strain revealed no evidence of integrated phage DNA, supporting a strictly lytic life cycle for all 3 phages. These observations are consistent with predictions from the BACPHLIP tool, which classified Spear, Loop, and Shorty as virulent with confidence scores of 0.9625, 0.9483, and 0.9625, respectively (Hockenberry and Wilke 2021). None of the phages encode tRNAs, and additional analysis against the VFDB confirmed the absence of known virulence factors that might enhance host bacterial fitness.

### Host–phage interaction indicates shared receptor usage

**Figure 3.**
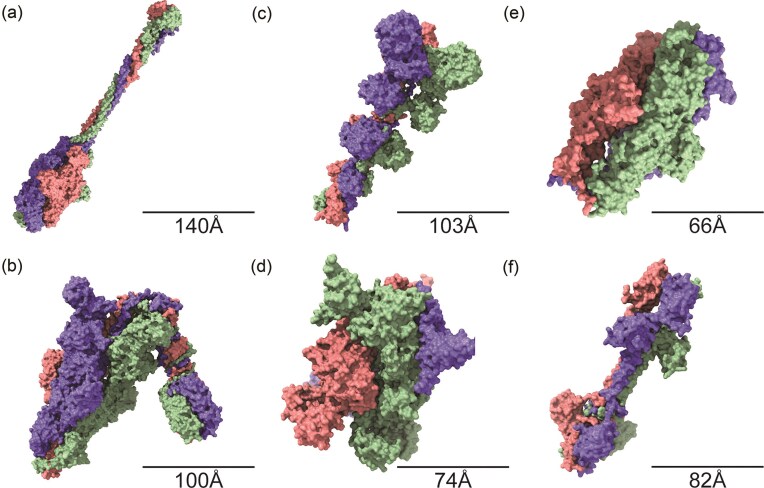
*In silico* models of six putative RBPs in their trimeric forms. RBPs of Loop (CDS_0007 and CDS_0009) (a, b), Spear (CDS_0005 and CDS_0006) (c, d) and Shorty (CDS_0002 and CDS_0014) (e, f) displayed by their surface zones. The colours denote three chains of a trimer. Dimensions computed in UCSF ChimeraX.

Since all three phages infect the same *K. pneumoniae* host, we sought to determine whether they, despite being genetically unrelated, recognize the same receptor and therefore possess similarly structured receptor-binding proteins (RBPs). RBPs are typically located within the tail fibers, tail spikes or baseplate of the phage and are crucial for host recognition. A total of six putative RBPs were identified, two for each phage. Given that previous studies have shown tail fiber and tail spike proteins to commonly assemble as trimers, we generated *in silico* models of the phage RBPs in their trimeric forms (Fig. 3) (Betts and King 1999, Garcia-Doval and van Raaij 2012, Islam et al. 2019).

The TEM of phage Spear previously revealed a distinctive, triangular appendage at its tail tip, visually reminiscent of a spearhead (Fig. 1a). This unique morphological feature measured ~27 nm across its longest dimension. Intriguingly, our structural modeling of the trimeric protein component (CDS_0005) of Spear predicted a molecular assembly with a maximal dimension of ~21 nm (Fig. 3c). This congruence in scale suggests that this trimeric protein could potentially form the structural basis of the observed spear-like feature at the tail tip of this particular phage.

Because all three phages exhibit a narrow host range limited to K1 serotype strains, we hypothesized that their RBPs might share either sequence or structural homology. DNA sequence analysis revealed no detectable homology between the corresponding RBP-encoding genes (Spear: CDS_0005; Spear: CDS_0006 Loop: CDS_0009; Shorty: CDS_0014) (Supp. Fig. S4). However, NCBI database comparisons revealed that on the amino acid level, a region of the Loop (CDS_0007) and Shorty (CDS_0002) proteins align, which cover positions 287–860 (Loop) and 73–648 (Shorty), respectively. Furthermore, we found these regions to align with a K1-specific lyase, which is consistent with the exhibited host range (Tu et al. 2022). Closer examination revealed that the RBPs from Loop (CDS_0007) and Shorty (CDS_0002) actually correspond to tail spike proteins, although they were annotated as tail fibers by the Pharokka tool. This functional similarity is further supported by the high degree of structural homology observed between the tail spikes from Loop and Shorty. Given the otherwise distinct genomic backbones of these phages, this observed homology in the K1-specific lyase domain suggests the possibility of genetic mosaicism, where these specific receptor-binding elements were acquired through horizontal gene transfer. That would also explain the presence of a halo within those two phages but not with Spear.

**Figure 4.**
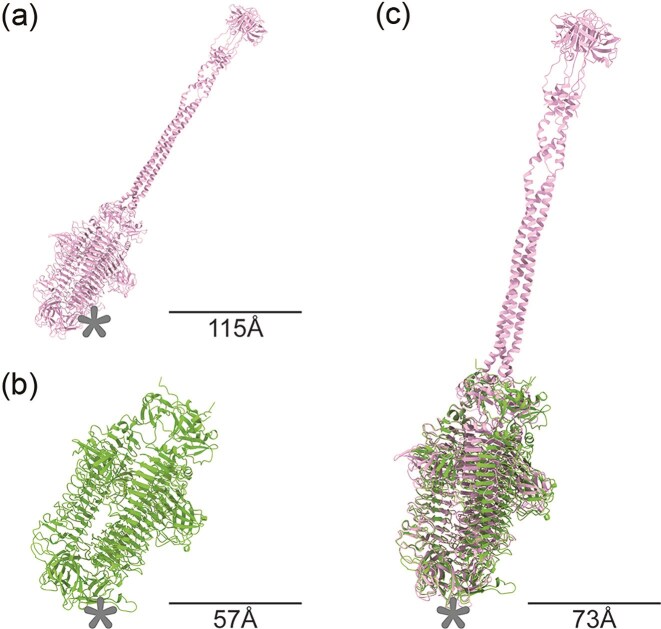
Tail spikes of Loop (a) and Shorty (b) modeled as trimers with secondary structures shown. Superimposed structures reveal a high degree of structural homology (c). Dimensions computed in UCSF ChimeraX. Asterisk indicates the predicted receptor-binding site location.

Additionally, the generated structures were utilized for structural overlay analysis to visualize and assess their similarity. Consistent with the prior HHPred homology search, only the tail spikes of Loop (CDS_0007) and Shorty (CDS_0002) have demonstrated structure homology in their multimeric forms. The two RBPs have a template modeling score (TM-Score) of 0.9238 when normalized to the length of a shorter trimer with an RMSD value of 1.87, suggesting a high degree of similarity. When superimposing both structures on top of each other (Fig. 4), the aligned regions again coincide with K1-specific lyase domain (Tu et al. 2022). This suggests that it is likely that both tail spikes not only target the K1 polymer, but also bind the same structural moiety.

### All phages bind CPS

**Figure 5.**
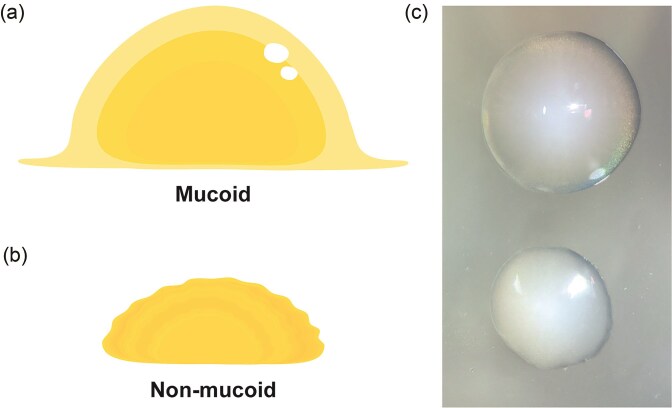
Schematic illustration of mucoid phenotype of the wild-type strain (a) and a loss of mucoidy in the resistant variants (b). Photograph of mucoid and non-mucoid colonies side-by-side taken at 20× magnification (c).

To determine whether K1 CPS serves as the receptor for both Loop and Shorty, 25 resistant variants were generated under selective pressure from Loop predation. All 25 colonies exhibited a loss of the hypermucoviscous phenotype characteristic of the wild-type strain (Fig. 5). In *K. pneumoniae*, the hypermucoid phenotype is typically associated with upregulation and overexpression of the CPS, particularly in K1 and K2 serotypes (Zhu et al. 2021). This observation suggests that selective pressure from Loop may have led to either downregulation of CPS synthesis or a loss-of-function mutation in one of the CPS biosynthesis genes.

**Figure 6.**
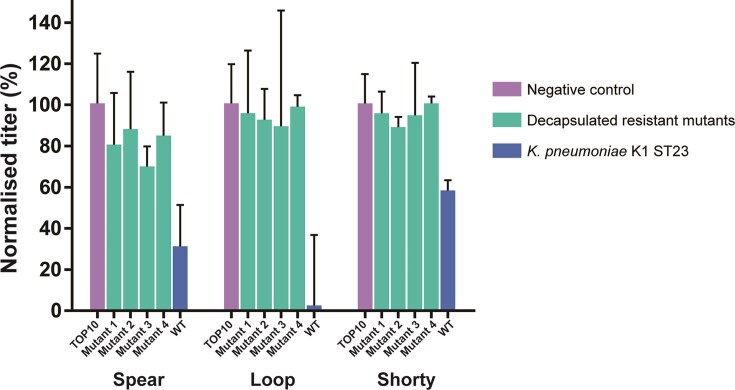
Max-normalized adsorption of Spear, Loop, and Shorty to wild-type host *K. pneumoniae* K1 ST23, 4 resistant mutants generated by Loop predation, and *E. coli* TOP10 cells as the negative control. Experiments were conducted in triplicate with 3 independent experiments. Error bars indicate standard deviation.

Cross-resistance experiments further revealed that Spear, Loop, and Shorty all exhibited either no infectivity or drastically reduced infectivity towards the resistant mutants. These findings suggest that all three phages likely depend on CPS for host recognition and subsequent infection. To determine whether the loss of hypermucoidy impaired phage binding or was a coincidental phenotypic change, we performed adsorption assay using all three phages on 4 randomly selected resistant variants (Fig. 6). The results show that all three phages exhibited markedly reduced binding to the decapsulated resistant mutants, compared to the wild-type strain.

For phages Loop and Shorty, adsorption to the resistant variants was comparable to the negative control strain (only 5.8% and 6.7% higher, respectively), suggesting that CPS may be their sole receptor (Fig. 6). In contrast, Spear retained partial binding to the decapsulated mutants (∼80% of the negative control), indicating that additional factors beyond the capsule may contribute to Spear adsorption.

Comparing binding to the wild-type strain, the three phages showed distinct binding efficiencies: Spear ∼31% unbound, Loop ∼2% unbound, and Shorty ∼58% unbound relative to the negative control. This is particularly surprising given that the tail spikes of Loop and Shorty share a high degree of structural homology, yet Loop binds the wild-type strain substantially more effectively, suggesting that regions beyond the homologous domain of Loop’s tail spike may contribute to CPS binding.

## Discussion

In this study, we characterized three distinct lytic bacteriophages, two siphovirus-like phages Spear and Loop, and the podovirus-like phage Shorty, all capable of infecting the same *K. pneumoniae* K1 ST23 strain, a hypervirulent lineage. Despite targeting the same host, the phages exhibit distinct morphological, genomic, and functional traits, supporting their classification as separate entities. Spear and Loop both display siphovirus morphology with non-contractile tails but differ markedly in plaque morphology, structural details, and RBP architecture. Notably, Loop forms halos around its plaques, indicative of depolymerase activity, which likely enhances its ability to penetrate the bacterial capsule (Beamud et al. 2023, Cheetham et al. 2024). Shorty is a podovirus-like phage that also forms halos around plaques, likely due to capsule-degrading depolymerases (Squeglia et al. 2020, Maciejewska et al. 2023). These enzymes remove the capsule barrier, allowing the phage to approach the membrane closely, which is crucial for genome translocation given its short tail. This is enabled by an ejectosome, intracapsid proteins that create a transmembrane DNA-tunnel upon infection (Leptihn, 2016, Swanson, 2021).

Although Spear and Loop share a siphovirus-like morphology, genomic analyses reveal no significant sequence homology between them; each aligns with different phages in the NCBI database. Although both phages encode anti-repressor genes, they lack hallmark temperate phage genes such as integrases and repressors. Consistently, whole-genome sequencing of the host strain showed no evidence of lysogeny, and BACPHLIP predicted all three phages as virulent with high confidence. These findings collectively confirm their strictly lytic life cycle, a desirable trait for therapeutic applications. Likewise, Shorty lacks lysogeny-associated genes. Furthermore, all three phages maintain infectivity at temperatures up to 50°C and demonstrate a narrow host range specific only to hypervirulent K1 ST23 strains. This specificity enhances their suitability as targeted phage therapy agents, potentially minimizing off-target effects on the native microbiota (Jończyk et al. 2011, Strathdee et al. 2023).

The initial step of phage infection involves specific binding to a host cell, a process mediated by phage ligand-binding proteins, commonly known as RBPs. Because our three phages infect the same bacterial host, they could employ distinct receptors or recognize a common one. We therefore investigated the structure of the RBPs in all three phages. Despite the fact that they share no sequence homology at the gene level, evaluating their structural homology revealed that a tail spike from Loop shows a high degree of structural similarity to a tail spike from Shorty, being almost identical. Their structural similarity is further supported by the observed sequence homology in the amino acid alignment of the receptor-binding domains with a known K1-specific lyase. This unexpected sequence homology within an otherwise genetically distinct phage background points towards genetic mosaicism, where the K1-specific receptor-binding domain was likely acquired independently by Loop and Shorty, or transferred from one to the other.

To explore receptor usage further, we isolated bacterial mutants resistant to all three phages, all of which displayed a non-mucoid phenotype indicative of capsule loss or downregulation. These results strongly suggest that CPS serves as the primary receptors or co-receptor for phage binding and infection. This shared reliance on CPS presents both an opportunity and a challenge: while targeting a conserved and essential virulence factor increases therapeutic efficacy, it may also drive resistance through capsule loss or modification. Importantly, such resistance frequently incurs a fitness cost, as evidenced by the loss of mucoidy, which may attenuate virulence *in vivo* (Wang, 2021, Zierke et al. 2025). This loss of a key virulence factor, whether CPS, LPS, or an outer membrane protein, is often observed to reduce the pathogen’s ability to colonize, evade the immune system, or cause severe disease. For Spear, CPS might not be the sole receptor, although it remains critical for efficient infection. This partial divergence in receptor usage could explain the observed differences in binding efficiency and resistance profiles.

In conclusion, our findings illustrate a compelling example of shared receptor usage among genetically unrelated phages targeting the same bacterial structure, potentially driven by genetic mosaicism. These insights deepen our understanding of phage–host interactions and provide a foundation for the rational design of CPS-targeting phage therapies. This shared receptor dependence, however, implies that one has to be careful when designing a phage cocktail. Relying on phages of different morphologies and unrelated genetic backgrounds might not be sufficient, as this might lead to a cocktail composed of phages relying on the same primary receptor, regardless of their family or genomic diversity. These phages would be rendered entirely ineffective by a single bacterial resistance mutation affecting that common target. Therefore, a comprehensive understanding of specific receptor interactions is paramount to building robust cocktails that can withstand bacterial adaptation. Ultimately, our findings advocate for a shift from empirical phage selection to a more rational, receptor-guided approach for developing robust phage therapeutics against prevalent pathogens like hypervirulent *K. pneumoniae*.

## Supplementary Material

xtaf014_Supplemental_Files

## Data Availability

The sequence data have been deposited in the National Center for Biotechnology Information (NCBI) database. The GenBank Accession Numbers are: PX117328 for Spear, PX117329 for Loop and PX117330 for Shorty.
