# Supplementary material for: Deciphering shared receptor usage in genomically unrelated bacteriophages infecting hypervirulent Klebsiella pneumoniae K1 ST23
Source: FEMS Microbes. 2025 Oct 20;6:xtaf014. doi: 10.1093/femsmc/xtaf014 (PMC12611254; doi:10.1093/femsmc/xtaf014)
Supplement: xtaf014_Supplemental_Files [file xtaf014_supplemental_files.zip › FEMSMC-2025-030.R1 one sentence summary.docx]

Capsular polysaccharide is identified as a shared receptor among diverse bacteriophages infecting Klebsiella pneumoniae K1 ST23, offering new insights for rational phage therapy design.
