# Supplementary figures and images for: Deciphering shared receptor usage in genomically unrelated bacteriophages infecting hypervirulent Klebsiella pneumoniae K1 ST23
Source: FEMS Microbes. 2025 Oct 20;6:xtaf014. doi: 10.1093/femsmc/xtaf014 (PMC12611254; doi:10.1093/femsmc/xtaf014)

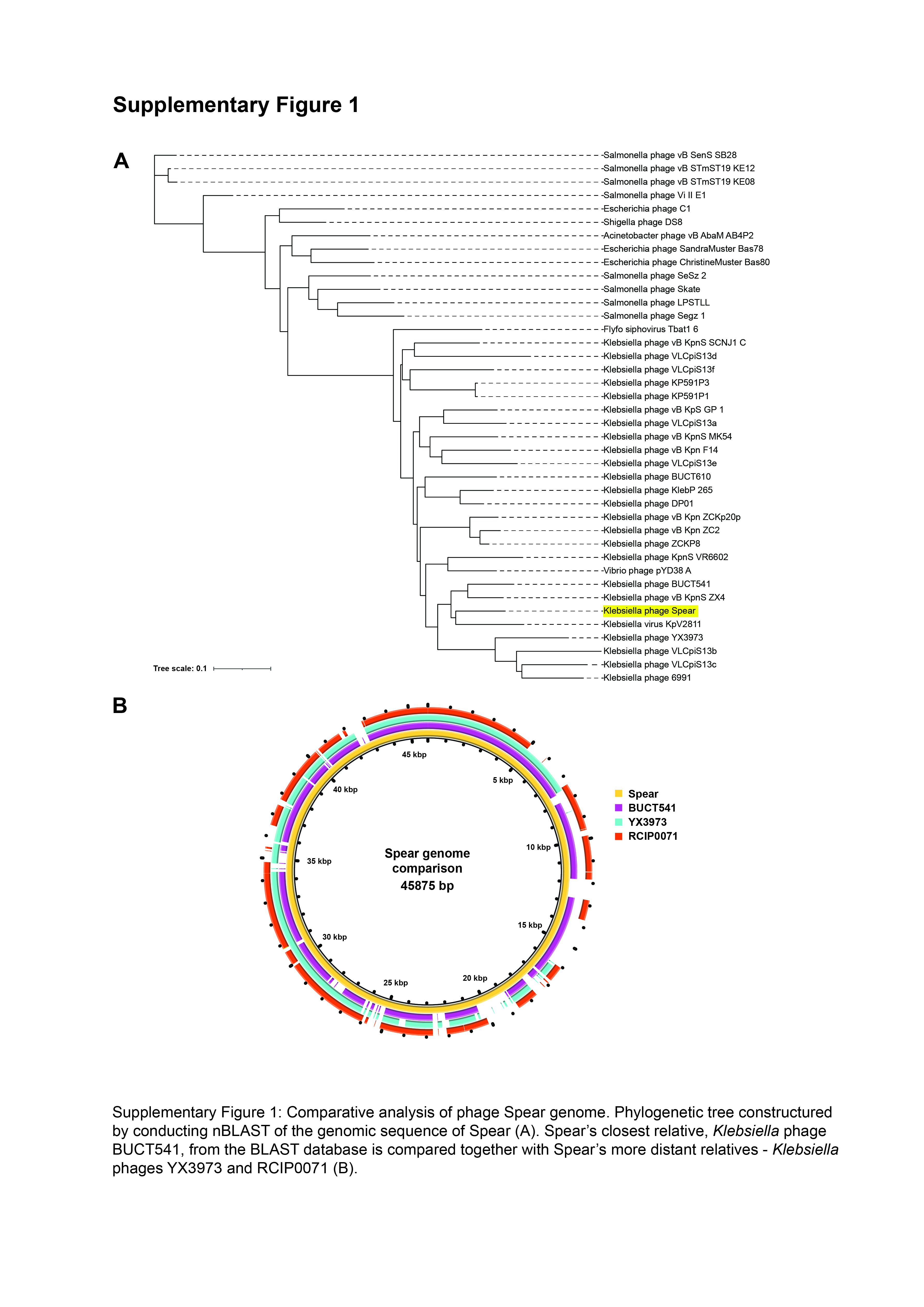

Supplement: xtaf014_Supplemental_Files [file xtaf014_supplemental_files.zip › SF1_alignArtboard 1@6x-100.jpg]

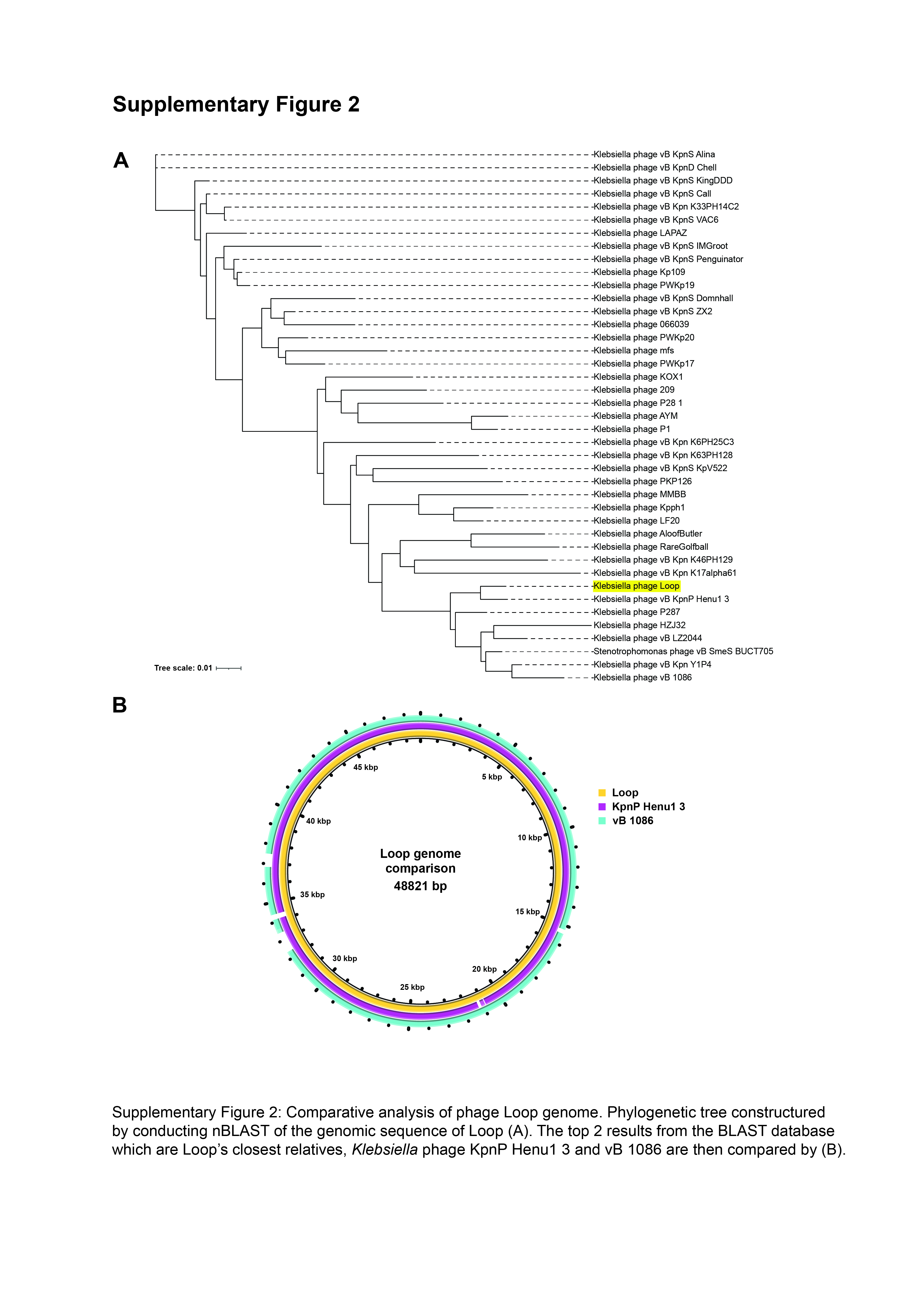

Supplement: xtaf014_Supplemental_Files [file xtaf014_supplemental_files.zip › SF2_alignArtboard 1@6x-100.jpg]

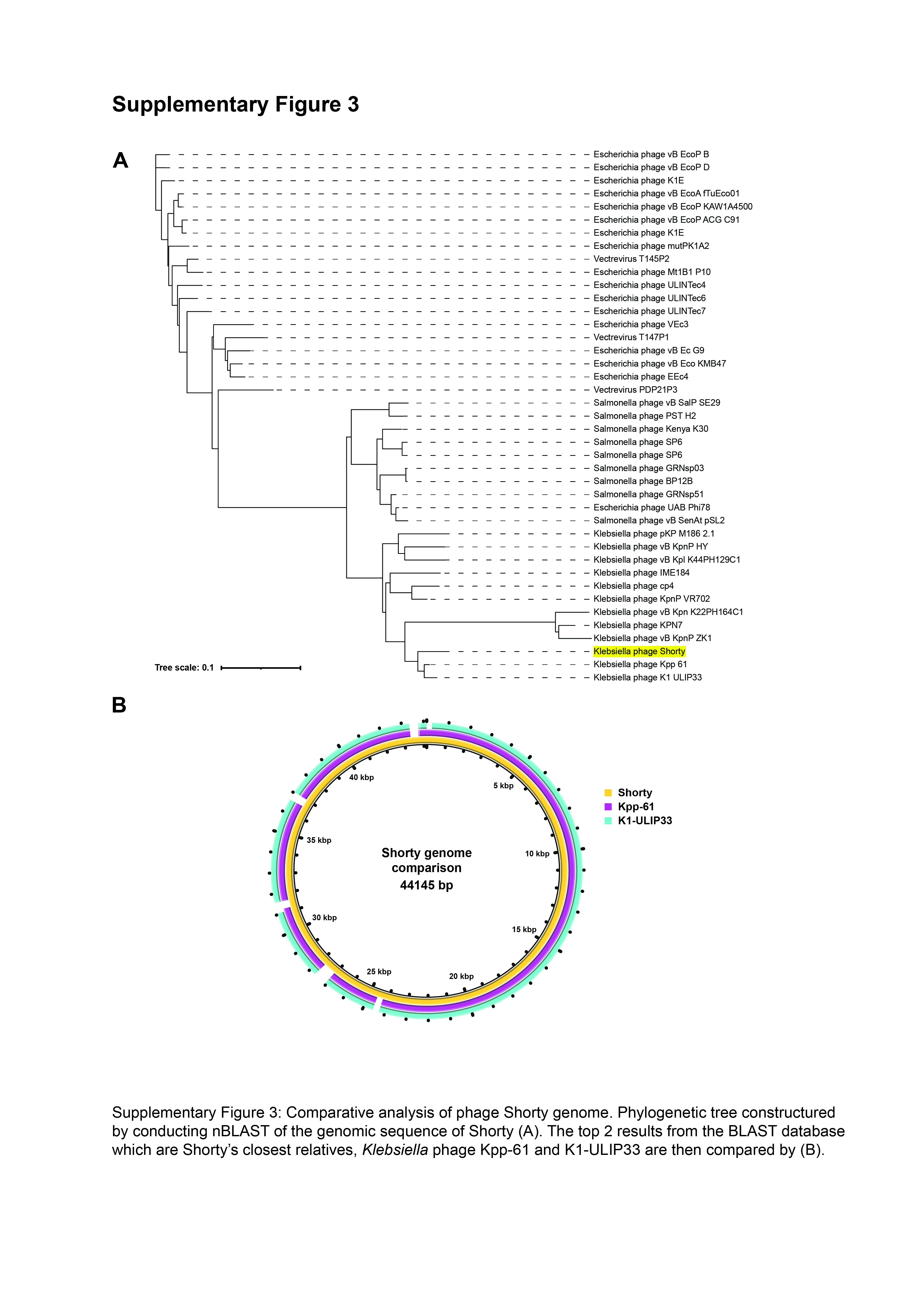

Supplement: xtaf014_Supplemental_Files [file xtaf014_supplemental_files.zip › SF3_alignArtboard 1@6x-100.jpg]

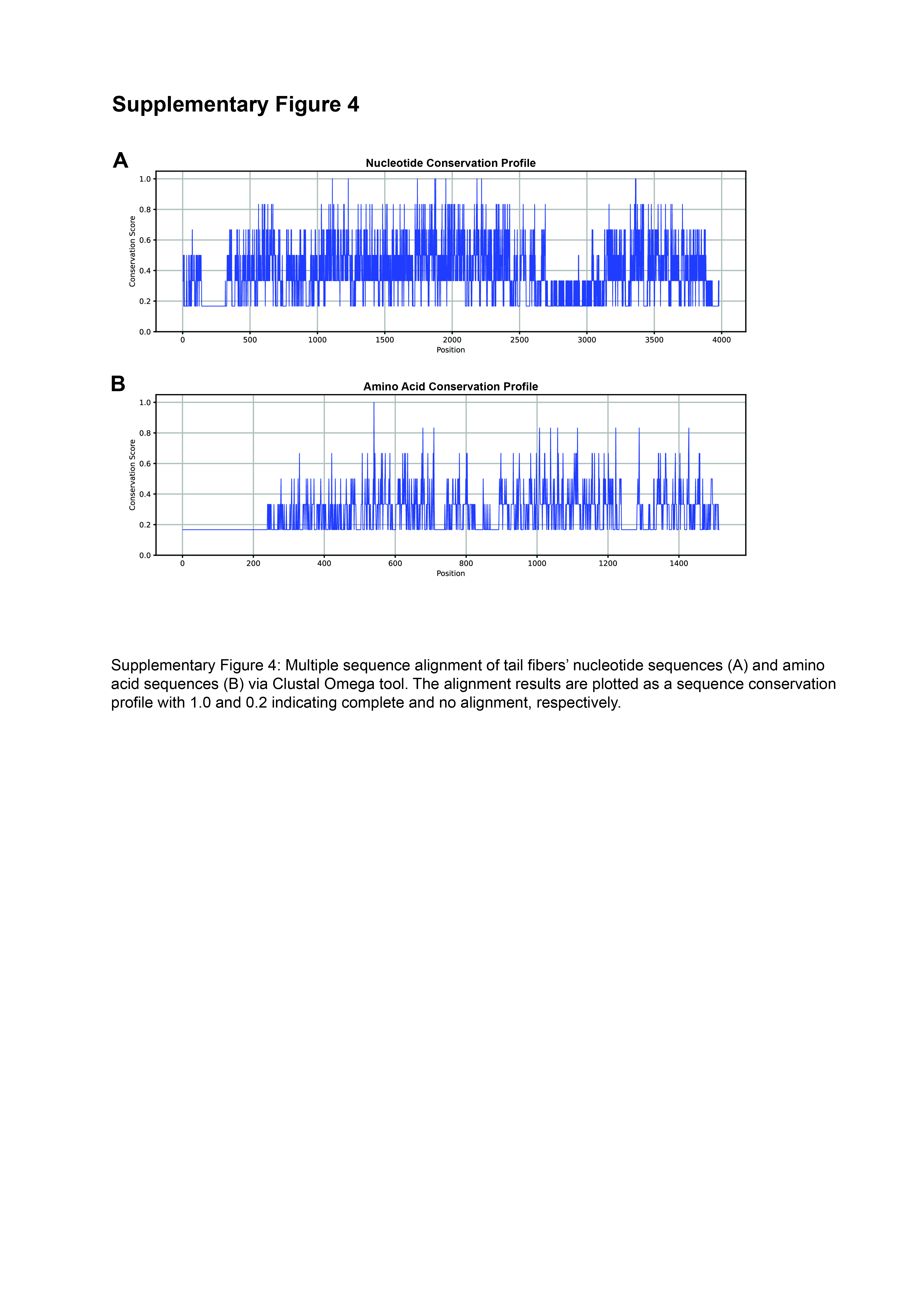

Supplement: xtaf014_Supplemental_Files [file xtaf014_supplemental_files.zip › SF4_alignArtboard 1@6x-100.jpg]
