# Supplementary material for: Deciphering shared receptor usage in genomically unrelated bacteriophages infecting hypervirulent Klebsiella pneumoniae K1 ST23
Source: FEMS Microbes. 2025 Oct 20;6:xtaf014. doi: 10.1093/femsmc/xtaf014 (PMC12611254; doi:10.1093/femsmc/xtaf014)
Supplement: xtaf014_Supplemental_Files [file xtaf014_supplemental_files.zip › FEMSMC-2025-030.R1 supplementary data - reviews and author response.docx]

**Reviews of manuscript FEMSMC-2025-030.r1**

**Reviewer 1**

**Recommendation: Minor Revision**

Minor suggestions:

Page 2. For the graphical abstract, place the names Loop, Spear, and Shorty above their respective cartoons.
Page 5, Line 24, briefly define hypermucoviscosity.

Page 6, Line 25, what is the name and characteristics of the Kpn isolation host?

Page 6, Line 40 “a 1:1 ratio” instead of “the 1:1 ratio.”

Page 7, Line 5, “had solidified” instead of “has solidified.”

Page 10, Lines 3-6, make clear that this lone K1 in the collection is not the isolation host of the three phages.

Page 11, Line 34, I think life cycle should be two words, as it is not common to see it as one.

Page 13, Lines 14 and 24: what are the mutations in the capsule null resistant strains? Are the 25 different from each other or clones? Particularly, what is the nature of those used in Figure 6. In the text it says 4 capsule strains, but the Figure only indicates one resistant mutant. Different capsule mutations can affect things like binding, so I think it is relevant to know what gene (or genes) were interrupted and if it is just one mutant or several.

Page 19, Figure 1. Include a scale bar on the photos of B, E, and H.

I enjoyed reading this well-written article describing Loop, Spear, and Shorty. My one concern is that we do not know the nature of the capsule mutant(s) used for Figure 6.

**Reviewer 2**

**Recommendation: Minor Revision**

The manuscript presents a thorough characterization of three newly isolated Klebsiella phages, highlighting their shared receptor usage and structural similarities. The authors combine phenotypic assays, adsorption experiments, genomic comparisons, and structural modelling to provide compelling evidence for the role of capsular polysaccharide as the primary receptor and for functional differences among related receptor-binding proteins.

The study is well designed, and the data are generally convincing. Figures are clear, and the methods are described in sufficient detail to ensure reproducibility. The link between receptor specificity, phage cocktail design, and therapeutic applications is timely and relevant.

Some minor issues should be addressed before final publication:

Ensure consistency in terminology (e.g., use “podovirus-like” throughout, unify “PFU/mL” and “× g” notations).

Clarify the adsorption assay results, as the current wording seems inconsistent with the quantitative data (Loop vs. Shorty binding).

Verify the accession numbers, as the PX-prefix may be a placeholder.

Improve figure legends with precise wording (e.g., dimensions “computed in UCSF ChimeraX,” indicate receptor-binding site location in Fig. 4).

Minor typographical and style corrections (e.g., E. coli, halos/haloes, min vs mins).

Overall, this is a solid and interesting manuscript that makes a valuable contribution to the understanding of phage–host interactions in Klebsiella. After minor revisions for clarity and consistency, it should be suitable for publication.

**Associate Editor Recommendation: Minor Revision**

Thank you for the privilege of reviewing your work. Your manuscript has now been reviewed my two experts in the field. Both are very enthusiastic about the study. However, some minor revisions are requested by both. Please do pay careful attention to reviewer 1's query about the nature of the capsule mutants if you choose to revise your manuscript.

**EIC Decision: Minor Revision**

**Author's Response to Decision Letter for (FEMSMC-2025-030)**

We would like to thank the reviewers for their time and for the comments to help improve our manuscript for publication. Below, we provide point-by-point responses to each reviewer comment, with corresponding manuscript changes indicated.

Reviewer: 1

> Comments to the Author

> I enjoyed reading this well-written article describing Loop, Spear, and Shorty. My one concern is that we do not know the nature of the capsule mutant(s) used for Figure 6. Please see attached file for minor suggestions.

We thank the reviewer for the positive feedback.

We address the concern about the capsule mutants below (directed at Page 13, Lines 14 and 24).

> Minor suggestions:

> Page 2. For the graphical abstract, place the names Loop, Spear, and Shorty above their respective cartoons.

We agree and have now included the names in the graphical abstract.

> Page 5, Line 24, briefly define hypermucoviscosity.

Agreed. We have now included a brief definition:“Hypermucoviscosity is characterised by an overexpression of capsular polysaccharide (CPS) on the surface of bacteria which protects bacterial communities from both antibiotic efficacy and host immune responses.” This is found in line 5 of paragraph 2 in the Introduction.

> Page 6, Line 25, what is the name and characteristics of the Kpn isolation host?

We have added this information. “The isolation host (Kp290-019) is a hypermucoid and hypervirulent strain of ST23 K1 lineage.”

> Page 6, Line 40 “a 1:1 ratio” instead of “the 1:1 ratio.”

We agree and have made this change.

> Page 7, Line 5, “had solidified” instead of “has solidified.”

We agree and have made this change.

> Page 10, Lines 3-6, make clear that this lone K1 in the collection is not the isolation host of the three phages.

We thank the reviewer for pointing this out. We have now changed the sentence to: “out of 48 K. pneumoniae strains tested via duplicate top agar overlay assays, all three phages infected both K. pneumoniae K1 ST23, O1ab serotype strains in our collection, the original isolating host and one other hypervirulent strain, indicating an extremely narrow host range.”

> Page 11, Line 34, I think life cycle should be two words, as it is not common to see it as one.

We agree and have changed this.

> Page 13, Lines 14 and 24: what are the mutations in the capsule null resistant strains? Are the 25 different from each other or clones? Particularly, what is the nature of those used in Figure 6. In the text it says 4 capsule strains, but the Figure only indicates one resistant mutant. Different capsule mutations can affect things like binding, so I think it is relevant to know what gene (or genes) were interrupted and if it is just one mutant or several.

We thank the reviewer for this insightful comment. All 25 strains are distinct from each other as they have all emerged independently as separate colonies during their generation, which was further confirmed by sequencing. The reviewer is correct that Figure 6 included only one resistant mutant while the text referred to 4. The revised Figure 6 now presents data for all 4 resistant mutants, consistent with the text. Sequencing of these 4 mutants tested revealed mutations in genes responsible for capsule biosynthesis and export. While the specific genetic disruptions vary, the consistent outcome across all mutants is the decapsulated phenotype, which is the critical feature relevant to the adsorption assays.

We agree that detailed genetic characterisation of these mutants is important, and a comprehensive analysis will be included in a forthcoming manuscript currently in preparation.

> Page 19, Figure 1. Include a scale bar on the photos of B, E, and H.

We agree and have included a scale bar to each image.

> Reviewer: 2

> Comments to the Author

> The manuscript presents a thorough characterization of three newly isolated Klebsiella phages, highlighting their shared receptor usage and structural similarities. The authors combine phenotypic assays, adsorption experiments, genomic comparisons, and structural modelling to provide compelling evidence for the role of capsular polysaccharide as the primary receptor and for functional differences among related receptor-binding proteins.

> The study is well designed, and the data are generally convincing. Figures are clear, and the methods are described in sufficient detail to ensure reproducibility. The link between receptor specificity, phage cocktail design, and therapeutic applications is timely and relevant.

We thank the reviewer for the kind and encouraging comments.

> Some minor issues should be addressed before final publication:

> Ensure consistency in terminology (e.g., use “podovirus-like” throughout, unify “PFU/mL” and “× g” notations).

We agree and have changed all variations to “podovirus-like”. We have also unified “PFU/mL” and “x g” notations throughout the manuscript.

> Clarify the adsorption assay results, as the current wording seems inconsistent with the quantitative data (Loop vs. Shorty binding).

We thank the reviewer for pointing out the inconsistency. We have clarified the adsorption assay results in the text by explicitly quantifying binding of each phage to the resistant mutants and the wild-type strain, and by highlighting the differences between Loop and Shorty binding. These changes clarify that Loop and Shorty show minimal binding to decapsulated mutants, whereas Spear retains partial binding.

> Verify the accession numbers, as the PX-prefix may be a placeholder.

NCBI GenBank provided PX-numbers as accession numbers – we can confirm this by providing the confirmation email from GenBank. The accession numbers were issued on August 13, 2025.

> Improve figure legends with precise wording (e.g., dimensions “computed in UCSF ChimeraX,” indicate receptor-binding site location in Fig. 4).

We agree and have improved wording in the figure legends as suggested.

> Minor typographical and style corrections (e.g., E. coli, halos/haloes, min vs mins).

We agree and have standardized style.

> Overall, this is a solid and interesting manuscript that makes a valuable contribution to the understanding of phage–host interactions in Klebsiella. After minor revisions for clarity and consistency, it should be suitable for publication.

**Reviews of manuscript FEMSMC-2025-030.R1**

**Associate Editor Recommendation: Accept**

**EIC Decision: Accept**
